# Clinical study on primary screening of oral cancer and precancerous lesions by oral cytology

**DOI:** 10.1186/s13000-020-01027-6

**Published:** 2020-09-10

**Authors:** Shintaro Sukegawa, Sawako Ono, Keisuke Nakano, Kiyofumi Takabatake, Hotaka Kawai, Hitoshi Nagatsuka, Yoshihiko Furuki

**Affiliations:** 1 Department of Oral and Maxillofacial Surgery, Kagawa Prefectural Central Hospital, 1-2-1, Asahi-machi, Takamatsu, Kagawa 760-8557 Japan; 2 Department of Oral Pathology and Medicine, Okayama University Graduate School of Medicine, Dentistry and Pharmaceutical Sciences, Okayama, Japan; 3 Department of Pathology, Kagawa Prefectural Central Hospital, Takamatsu, Kagawa Japan; 4 Department of Pathology, Okayama University Graduate School of Medicine, Dentistry, and Pharmaceutical Sciences, Okayama, Japan

**Keywords:** Cytology, Pathology, Liquid-based cytology, Screening, Inflammation

## Abstract

**Background:**

This study was conducted to compare the histological diagnostic accuracy of conventional oral-based cytology and liquid-based cytology (LBC) methods.

**Methods:**

Histological diagnoses of 251 cases were classified as negative (no malignancy lesion, inflammation, or mild/moderate dysplasia) and positive [severe dysplasia/carcinoma in situ (CIS) and squamous cell carcinoma (SCC)]. Cytological diagnoses were classified as negative for intraepithelial lesion or malignancy (NILM), oral low-grade squamous intraepithelial lesion (OLSIL), oral high-grade squamous intraepithelial lesion (OHSIL), or SCC. Cytological diagnostic results were compared with histology results.

**Results:**

Of NILM cytology cases, the most frequent case was negative [LBC *n* = 50 (90.9%), conventional *n* = 22 (95.7%)]. Among OLSIL cytodiagnoses, the most common was negative (LBC *n* = 34; 75.6%, conventional *n* = 14; 70.0%). Among OHSIL cytodiagnoses (LBC *n* = 51, conventional *n* = 23), SCC was the most frequent (LBC *n* = 31; 60.8%, conventional *n* = 7; 30.4%). Negative cases were common (LBC *n* = 13; 25.5%, conventional *n* = 14; 60.9%). Among SCC cytodiagnoses SCC was the most common (LBC *n* = 16; 88.9%, conventional *n* = 14; 87.5%). Regarding the diagnostic results of cytology, assuming OHSIL and SCC as cytologically positive, the LBC method/conventional method showed a sensitivity of 79.4%/76.7%, specificity of 85.1%/69.2%, false-positive rate of 14.9%/30.7%, and false-negative rate of 20.6%/23.3%.

**Conclusions:**

LBC method was superior to conventional cytodiagnosis methods. It was especially superior for OLSIL and OHSIL. Because of the false-positive and false-negative cytodiagnoses, it is necessary to make a comprehensive diagnosis considering the clinical findings.

## Background

Head and neck cancer is one of common malignancies in the world, and the most common histopathological type is squamous cell carcinoma (SCC). Several patients die every year because of advanced oral SCC [1]. Conversely, studies have reported that early detection and treatment of oral SCC can reduce mortality and morbidity and increase the likelihood of complete recovery [2, 3]. Therefore, it is important to use the simplest and the most accurate method that can to detect early-stage abnormalities in oral mucosal cells. An example of such method is exfoliated mucosal cytology, which involves making a diagnosis from minimally invasive oral mucosal cells [4].

Exfoliative cytology is the microscopic examination of shed or desquamated cells from the mucus membrane, and it is a simple, safe, and reliable approach. Exfoliative cytology consists of the conventional method and the liquid-based cytology (LBC) method [5]. The conventional method involves chair side scraping of the oral mucosa and then smearing it directly onto a glass slide. This method requires a suitable technique because the morphology of the collected cells would change if handled improperly. Conversely, LBC is a technique in which cells are scattered in a fixative liquid to produce a thin layer of cells on the slide. Therefore, the LBC method has been widely used because of its advantage of not requiring complicated operations on the chair side.

However, the current gold standard for diagnosing oral epithelial dysplasia and cancer is not exfoliative excision cytology; rather, it is resection biopsy or histological examination of surgical specimens [6, 7]. Unfortunately, excision biopsy is an invasive diagnostic method, and scrape cytology is suitable for the screening of pathological conditions considering minimal invasiveness. However, the diagnostic capabilities of two types of oral exfoliative excision cytology, the conventional and LBC methods, remain unclear. Furthermore, the diagnostic accuracy of the oral scraping cytology compared with the histopathological diagnosis has not been examined in detail. Therefore, it is important to consider whether the two types of cytology are sufficient to be used as a standard method for the diagnoses of suspicious oral lesions.

This study was conducted to compare the histological diagnostic accuracy between the conventional method and the LBC method and to clarify the effectiveness of cytology.

## Methods

### Study design and sample

We designed and implemented a cross-sectional study using the oral exfoliative cytology results of patients who had been referred to the Kagawa Prefectural Central Hospital (Takamatsu, Japan) for diagnosis, treatment, and examination of oral lesions. During the period from April 2010 to March 2019, a total of 1234 specimens were obtained from the cytology specimens collected by the Department of Oral and Maxillofacial Surgery and diagnosed by the Department of Pathology. Of these specimens, nine were insufficient and excluded. Of the remaining 1225 specimens, 251 specimens that underwent histological diagnosis ranging from benign or malignant oral lesions for biopsy and/or surgical resection were included in this study. All specimens were collected, processed, and diagnosed in a single general hospital.

Cytology specimens were processed by conventional cytology from April 2010 to March 2015 and by the LBC method from April 2015 to March 2019. Cells were harvested by scraping with a cotton cytobrush device in all cases. In the conventional method, the collected cells were smeared onto a glass slide to prepare a sample, immersed in 95% ethanol, fixed, and stained with Papanicolaou stain. The LBC method involves dipping a cotton brush containing the sample directly into the transport medium, which is an alcohol-based preservative (BD CytoRich blue preservative, BD Japan, Tokyo, Japan). The liquid-based cellular material in the vial was processed according to the manufacturer’s protocol (BD Japan). The processing steps included vortexing of the sample, density reagent centrifugation, decantation and resuspension of cell pellets followed by gravity sedimentation on poly-l-lysine coated slides and subsequent staining with the Papanicolaou stain.

#### Procedure of cytological diagnosis

The specimens were reviewed by raters who had passed the board examination for cytology of the Japanese Society of Clinical Cytology. Cytology diagnostic experts confirmed that the sample was appropriate for cytology diagnosis. According to the criteria for specimen adequacy, we identified non-diagnosable specimens as inappropriate due to the presence of hypocellular or air-drying artifacts. The specimens were evaluated independently by at least two raters, and a representative cytology result of each case was determined by a majority vote. Cytological diagnoses were made based on the Bethesda system according to the Japanese society of clinical cytology (JSCC) diagnostic guideline and were classified into negative for intraepithelial lesion or malignancy (NILM), oral low-grade squamous intraepithelial lesion (OLSIL), oral high-grade squamous intraepithelial lesion (OHSIL), SCC, and indefinite for neoplasia [8].

#### Procedure of histological diagnosis

A histological diagnosis was provided by pathologists, and then, the number of biopsy samples was determined at the investigator’s discretion. These histological slides were subjected to hematoxylin and eosin staining, and their histological findings were divided into two categories as negative group and positive group. Negative was defined as non-malignant lesions, including inflammatory ones and mild, or moderate dysplasia. Positive was defined as severe dysplasia, carcinoma in situ (CIS), SCC, and other malignancies. Histological diagnosis was based on the WHO criteria [9]. CIS was in accordance with the general rules for clinical and pathological studies on oral cancer [10].

The design and methodology of this study have been approved by the Ethics Committee of Kagawa Prefectural Central Hospital (Approval No. 946).

### Statistical analysis

Data were entered into a database using Microsoft Excel (Microsoft Inc., Redmond, WA, USA). The database was transferred to JMP version 14.2 for Macintosh computers (SAS Institute Inc., Cary, NC, USA) for statistical analysis. To compare between cytological and histological diagnoses, the histological diagnoses were classified into negative and positive, and the cytological diagnoses were also classified into negative (NILM and OLSIL) and positive (OHSIL, SCC, and other malignancies). The diagnostic performance metrics was examined by comparing the cytological diagnosis against the histological diagnosis, for which the sensitivity, specificity, accuracy, positive predictive value, and negative predictive value were calculated, followed.

a $$ \mathrm{Sensitivity}=\frac{\mathrm{TP}}{\mathrm{TP}+\mathrm{FN}} $$

b $$ \mathrm{Specificity}=\frac{\mathrm{TN}}{\mathrm{TN}+\mathrm{FP}} $$

c $$ \mathrm{Accuracy}=\frac{\mathrm{TP}+\mathrm{TN}}{\mathrm{TP}+\mathrm{FP}+\mathrm{TN}+\mathrm{FN}} $$

d $$ \mathrm{Positive}\ \mathrm{predictive}\ \mathrm{value}=\frac{\mathrm{TP}}{\mathrm{TP}+\mathrm{FP}} $$

e $$ \mathrm{Negative}\ \mathrm{predictive}\ \mathrm{value}=\frac{\mathrm{TN}}{\mathrm{TN}+\mathrm{FN}} $$

TP and TN indicate true positive and true negative classifications, respectively; FP and FN indicate false-positive and false-negative classifications, respectively.

## Results

### Rate of inappropriate cytological specimens

In this study, there were three cases (3.5%) of inappropriate cytological specimens in the conventional method. In the LBC method, there were no cases of insufficient sample processing.

### Histological diagnosis

The histological diagnoses of 251 cases were classified as negative and positive using both the LBC method and the conventional method (Table 1).

**Table 1:** Histopathological categories

Lesion	LBC (***n*** = 169)	Conventional (***n*** = 82)
**Histological negative cases**	*n* = 103	*n* = 53
Benign tumor	7	11
Inflammation	24	3
Leukoplakia	19	6
Lichen planus	6	4
Others	42	27
Mild Dysplasia	4	
Moderate dysplasia	1	2
**Histological positeve cases**	*n* = 66	*n* = 29
Severe dysplasia	5	1
CIS	6	2
SCC	55	26

“Others” includes no malignancy, epulis, and mucosel

### Comparison of LBC and conventional cytological diagnoses with histological diagnoses

Table 2 shows the distribution of histological diagnoses from the viewpoint of cytological diagnosis. Of the NILM cytology cases (78 in total, including 55 using the LBC method and 23 using the conventional method), the most frequent case was negative (50 cases via LBC; 90.9%, 22 cases via the conventional method; 95.7%). Among OLSIL cytodiagnosis (total 65, including 45 using the LBC method and 20 using the conventional method), the most common case was negative (LBC *n* = 34; 75.6%, conventional *n* = 14; 70.0%). Conversely, among the OHSIL cytology cases (total 74, including 51 using the LBC method and 23 using the conventional method), SCC (LBC *n* = 31; 60.8%, conventional *n* = 7; 30.4%) was the most frequent and negative cases (LBC *n* = 13; 25.5%, conventional *n* = 14; 60.9%) were common. Among the SCC cytology cases (total 34, including 18 using the LBC method and 16 using the conventional method), SCC (LBC *n* = 16; 88.9%, conventional n = 14; 87.5%) was the most common (Table 2).

**Table 2:** Results of cytological diagnoses in comparison with histopathological diagnoses

Cytological diagnosis	Positve		Negative		Total
	SCC	CIS/severe dysplasia	Mild/Moderate dysplasia	Negative	
Histopathological diagnosis (LBC)					
NILM	2	1	2	50	55
OLSIL	6	3	2	34	45
OHSIL	31	6	1	13	51
SCC	16	1	0	1	18
Total	55	11	5	98	169
Histopathological diagnosis (Conventional)					
NILM	1	0	0	22	23
OLSIL	4	1	1	14	20
OHSIL	7	1	1	14	23
SCC	14	1	0	1	16
Total	26	3	2	51	82

### Diagnostic performance of cytological diagnoses

The positive predictive value of each cytological diagnosis made using the LBC method and the conventional method is shown (Table 3). NILM and SCC demonstrated a high diagnostic accuracy with both methods. However, the positive predictive value of OHSIL for histological lesions was 34.8% in the conventional method but 72.5% in the LBC method.

**Table 3:** Diagnostic accuracy of LBC and conventional methods

Cytological diagnosis	Positive	Negative	Total	Positive predictive value (%)
Histopathological diagnosis (LBC)				
NILM	3	52	55	94.5
OLSIL	9	36	45	80.0
OHSIL	37	14	51	72.5
SCC	17	1	18	94.4
Less than OHSIL	12	88	100	88.0
OHSIL and above	54	15	69	78.3
Histopathological diagnosis (Conventional)				
NILM	1	22	23	95.7
OLSIL	5	15	20	75.0
OHSIL	8	15	23	34.8
SCC	15	1	16	93.8
Less than OHSIL	6	37	43	86.0
OHSIL and above	23	16	39	59.0

Regarding the diagnostic results of cytology, assuming that OHSIL and SCC were cytologically positive, the LBC method and the conventional method showed a sensitivity of 81.8 and 79.3%, a specificity of 85.4 and 69.8%, a false-positive rate of 14.6 and 30.2%, and a false-negative rate of 18.2 and 20.7%, respectively (Table 4).

**Table 4:** Diagnostic performance metrics of cytological diagnoses

Perfomance metrics	LBC mehod	Conventional method
Sensitivity	81.8%	79.3%
Specificity	85.4%	69.8%
Accuracy	84.0%	73.2%
False positive	14.6%	30.2%
False negative	18.2%	20.7%

### False-negative cases

**Fig. 1:**
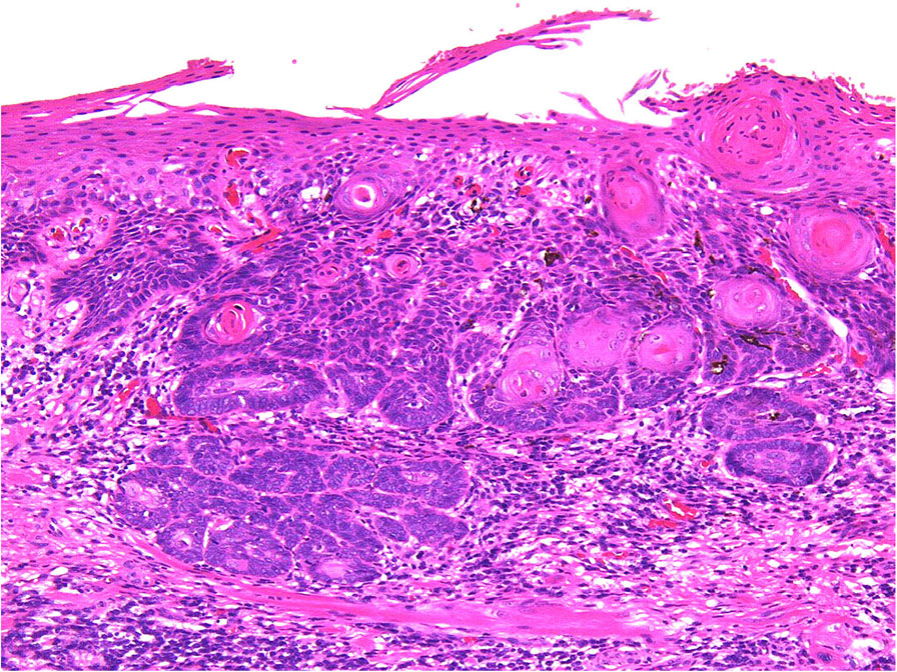
False-negative representative cases. The deep part of the epithelium reveals downward growth and invasion of tumor cells in the underlying tissue, and surface layer of the epithelium shows keratinocytes without prominent atypia

There were false-negative 12 cases in the LBC method. The cytology diagnoses were nine OLSIL cases and three NILM cases, and the histological types were eight SCC cases, two CIS cases, and two severe dysplasia cases. There were six false-negative cases in the conventional method. The cytological diagnoses were five OLSIL cases and one NILM case, and the histological types were five SCC cases and one CIS case (Fig. 1).

## Discussion

The results of our study demonstrate that the LBC method is superior to the conventional method for cytodiagnosis. The LBC method was especially superior for OLSIL and OHSIL for the positive predictive value. Conversely, because of a certain number of false-positives and false-negatives in cytodiagnosis, it is important to make a comprehensive diagnosis considering the clinical findings.

In this study, there were three cases of inappropriate cytological specimens in the conventional method. Sekine et al. [7] reported that 22.7% of unsuitable specimens were produced by the conventional smearing technique. They reported that the unsuitable samples in the conventional method were those that were considered to be inadequate for cytodiagnosis due to strong cell or air-drying artifacts. Our inappropriate specimens were similar, but the number of inappropriate specimens was very small in our study. We considered that this small number of inappropriate specimens occurred as the slides were created on the chair side by a specialist cytology technician when preparing samples using the conventional method. Conversely, in the LBC method, there were no cases of insufficient sample processing, and stable sample preparation was possible. This is consistent with previous reports showing that the LBC method obviously results in fewer inappropriate specimens [11]. Thus, it is important to understand and standardize the features and equipment of cytology collection methods to improve the quality of cytology slides.

Regarding the diagnostic efficacy of the methods, the LBC method has been demonstrated to be more effective than the conventional method [12]. Although past study have reported higher sensitivity (conventional/ LBC;85.7%/ 95.1%) and specificity (conventional/ LBC;95.9%/ 99.0%)for the LBC method than for the conventional method [6], another study reported that sensitivity (conventional/LBC; 96.3%/97.5%) of the LBC method was particularly good, whereas its specificity (conventional/LBC; 90.6%/68.7%) was reduced [13]. Our results indicated an increase in both sensitivity (conventional/LBC; 79.3%/81.8%) and specificity (conventional/LBC; 69.8%/85.4%). As the results were judged using the same criteria at the same facility, they were valuable demonstrating the usefulness due to differences in cytodiagnosis preparation. Conversely, there are also some very interesting results in our study. The positive predictive value was not different for NILM and SCC between the conventional method and the LBC method. However, a large difference was observed in the positive predictive value between OLSIL and OHSIL. Especially for OHSIL, it was a large difference. We speculate that this is due to a decrease in the number of negative cases misclassified as OHSIL and an increase in the number of detections of CIS and severe dysplasia. The LBC method contributes to the detection without missing CIS and severe dysplasia. These diseases are susceptible to SCC and are eligible for treatment, and appropriate detection of these diseases is clinically useful.

Despite the improvement in efficacy with the LBC method, there were a certain number of false-positives and false-negatives in this study. There were 12 cases of false-negatives in the LBC method and five cases in the conventional method (false-negative rate; LBC method 18.2%, conventional method 20.7%). When we examined the histology of cases diagnosed as NILM or OLSIL but with SCC, all the histological diagnoses of SCC were found to be well-differentiated SCCs. In addition, all cases were accompanied with well-differentiated keratinocytes lacking strong atypia on the surface. In this study, whether a problem existed with the site of cytology collection cannot be examined. The current diagnostic criteria for the JSCC are limited for the evaluation of atypical superficial keratinocytes. However, even in keratinized epithelium, which is histologically atypical, cytology has the advantage of examining individual cells in detail. Therefore, it might be possible to detect highly differentiated tumor cells that exhibit a tendency to keratinize. The diagnostic criteria of the JSCC take this point into account, but further examination of the diagnostic criteria is probably necessary in the future [8]. Suzuki et al. reported that false-negative cytology was more likely to occur in cases where the exposed cell area for diagnosis was very small or where very limited growth was observed [14]. Because it was difficult to collect basal or parabasal-like atypical cells, cells useful for cytodiagnosis were not sampled, thus leading to false-negative results. Sekine et al. reported [7] a false-negative rate of 22.2%, and our result for oral scraping cytology was acceptable.

Unfortunately, we also detected some false-positives. Despite the diagnosis of SCC by the conventional method and the LBC method, the cytodiagnosis was incorrect in each case. Despite the cytological diagnosis of SCC, one negative case was found with the use of both the conventional and LBC methods. However, there were 14 and 15 negative cases of OHSIL with the use of the LBC and conventional methods, respectively (false-positive rate: 14.6% by the LBC method and 30.2% by the conventional method). Many atypical epithelium with large nuclei are often observed, and the presence of epithelium that is partly suspected to be regenerating epithelium is suspected to be a malignant tumor; however, this is often confirmed even in cases associated with inflammation such as ulcer margin or candida infection [15]. In this study as well, false-positives were observed because there were cases of OLSIL that had to be differentiated from SCCs.

According to Remmerbach [16], applying the LBC method instead of the conventional method slightly reduced the false-negative results but still left a significant number of false-negative results. This result was similar to our study results. The false-negative results of cytology may exacerbate an untreated carcinoma, which may not be further treated and followed up. The fact that the lesion may subsequently be fatally exacerbated from false-negative results implies that further improvement is required before oral cytology by itself becomes a completely reliable method. Reducing false-negatives in cytology diagnosis is more important. To that end, in cases that make the diagnosis difficult, “suspect” results tend to be considered as “positive” in terms of further action required. In the oral cavity where various conditions are mixed, there is a limit in oral cytology in which cells of only the surface layer are collected and diagnosed under a microscope. A diagnosis that comprehensively considers clinical information will be necessary.

This study being a single-institution cross-sectional research has some limitations, perhaps including a case bias. Conversely, cytodiagnostic technologists and doctors had established the diagnosis on the basis of the same diagnostic criteria; therefore, it would be better to compare the accuracy of the conventional method and the LBC method according to the technology in a future study. The second limitation of this study is the small number of cases. Although the results of histological examinations were correct, the number of histological examinations was small in cases with NILM diagnosis. Nevertheless, since only a few studies conducted till have compared histology and the number of cases, our study could be useful.

## Conclusion

This study demonstrates that the LBC method is superior to the conventional methods in that histological diagnosis is less discrepant with cytological classification. In particular, the LBC method has the advantage of reducing the number of misdiagnosed CIS and severe dysplasia cases in oral cytology. Conversely, because of a certain number of false-positives and false-negatives in cytodiagnosis, it is important to make a comprehensive diagnosis considering the clinical findings.

## Data Availability

The datasets generated and /or analyzed during the current study are not publicly available. The Ethics Committee review board restricts the use of the datasets to the current study only.

## Abbreviations

JSCC: Japanese society of clinical cytology
LBC: Liquid-based cytology
NILM: Negative for intraepithelial lesion or malignancy
OHSIL: Oral high-grade squamous intraepithelial lesion
OLSIL: Oral low-grade squamous intraepithelial lesion
SCC: Squamous cell carcinoma
